# Dental Composite Formulation Design with Bioactivity on Protein Adsorption Combined with Crack-Healing Capability

**DOI:** 10.3390/jfb8030040

**Published:** 2017-09-07

**Authors:** Chen Chen, Junling Wu, Michael D. Weir, Lin Wang, Xuedong Zhou, Hockin H. K. Xu, Mary Anne S. Melo

**Affiliations:** 1 State Key Laboratory of Oral Diseases, West China Hospital of Stomatology, Sichuan University, Chengdu 610041, China; Cchen@umaryland.edu (C.C.); zhouxd@scu.edu.cn (X.Z.); 2 Department of Prosthodontics, Endodontics and Operative Dentistry, University of Maryland School of Dentistry, Baltimore, MD 21201, USA; JWu@umaryland.edu (J.W.); MWeir@umaryland.edu (M.D.W.); LWang@umaryland.edu (L.W.); Hxu@umaryland.edu (H.H.K.X.); 3 Shandong Provincial Key Laboratory of Oral Biomedicine, Jinan 250012, China; 4 VIP Integrated Department, Stomatological Hospital of Jilin University, Changchun 130011, China; 5 Center for Stem Cell Biology & Regenerative Medicine, University of Maryland School of Medicine, Baltimore, MD 21201, USA; 6 Department of Mechanical Engineering, University of Maryland, Baltimore County, MD 21250, USA

**Keywords:** self-healing, microcapsules, mechanical property, protein repellent, dental composite

## Abstract

**Short Title:**

Protein-repellent dental composite with crack-healing ability

**Abstract:**

Fracture and secondary caries are the primary reasons for the failure of dental restorations. To face this omnipresent problem, we report the formulation design and synthesis of a protein-resistant dental composite composed of 2-methacryloyloxyethyl phosphorylcholine (MPC) that also can self-repair damage and recover the load-bearing capability via microencapsulated triethylene glycol dimethacrylate (TEGDMA) and *N*,*N*-dihydroxy ethyl-*p*-toluidine (DHEPT). The bioactivity of the resulting MPC-microencapsulated TEGDMA-DHEPT was evaluated on protein adsorption through early bacterial attachment. Its mechanical properties were also investigated, including self-healing assessment. Microcapsules of poly (urea-formaldehyde) (PUF) were synthesized by incorporating a TEGDMA-DHEPT healing liquid. A set of composites that contained 7.5% of MPC, 10% of microcapsules, and without MPC/microcapsules were also prepared as controls. The two distinct characteristics of strong protein repellency and load-bearing recovery were achieved by the combined strategies. The novel dual composite with a combination of protein-repellent MPC and PUF microcapsules for restoring microcracks is a promising strategy for dental restorations to address the two main challenges of fracture and secondary caries. The new dual composite formulation design has the potential to improve the longevity of dental restorations significantly.

## 1. Introduction

In a range of materials available for the restoration of the tooth cavity, composites are the predominantly selected choice as they offer advantages in their aesthetics and less invasive preparation techniques [1,2]. Prospective randomized controlled trials (RCTs) and systematic reviews have highlighted that even though the overall survival rates were satisfactory, there are high annual failure rates associated with composites [3,4]. The replacement of a failed composite restoration leads to an increase in cavity size and destruction of the remaining tooth structure [5,6]. Replacement costs represent an enormous annual expense in the United States, considering that the annual cost for tooth cavity restorations in the United States was $46 billion in 2005 [7].

The predominant reasons for composite restoration failures are secondary caries and restoration fractures, which represent more than 90% of recorded failures [8]. Short-term survival rates report secondary caries often occurred after three years or later, which contribute to the high annual failure rates related to this material [9]. Besides the lack of suitable mechanical properties reflected by fracture, the incidence rate for biological complications represented by secondary caries, with or without fracture of the restoration, has been reported to be close to twice as high as technical complications [9]. New rational materials design based on prior knowledge have been developed for finding advanced designs to address these ongoing problems of dental composites [10].

One step on this path addresses the inhibition of nonspecific adsorption of proteins at the surface of dental restorative materials [11]. Since protein adsorption is believed to be the first step in the salivary pellicle formation, which also leads to bacterial attachment and biofilm formation, the inhibition of this process is a potential target for antibacterial approaches. Among the many strategies for imparting high resistance to protein adsorption so far investigated, the polymer 2-Methacryloylothelxyethyl-phosphorylcholine (MPC), in particular, has been one of the most promising approaches [12,13]. Since proteins are hydrophobic, MPC shows a high resistance toward protein adsorption due to the low polymer–water interfacial energy and high hydrophilicity [14,15]. This feature plays a critical role in reducing protein adsorption and preventing the formation of any conditioning layer that might otherwise enable the bacteria to gain anchorage to the surface [15,16]. The excellent biocompatibility of MPC-containing polymers has also been confirmed [17]. To date, recently resin-based direct restorative materials modified with MPC have shown lower protein adsorption, which has been associated with oral bacterial reduction [18,19,20,21,22,23].

Given the major requirements of clinical services and materials after fracture, an autonomous crack-healing ability has recently captured a lot of attention due to the recovery strength of biomaterials after being forced to break [24]. This approach employs a liquid healing agent encapsulated in a polymeric shell to form microcapsules, which are then incorporated into a matrix material [25]. When cracking occurs, the propagating crack will rupture the microcapsules, release the healing liquid that has its polymerization trigger when in contact with capsules, and lead to the healing of the composite [26,27]. More recently, novel self-healing poly (urea-formaldehyde) (PUF) microcapsules containing polymerizable TEGDMA and *N*,*N*-dihydroxy ethyl-*p*-toluidine (DHEPT) were synthesized and incorporated into dental resin good self-healing efficacy [28,29].

A combination of adequately selected strategies can bring synergistic effect that can help to overcome the two most predominant reasons for failures in composite restorations. The design and development of a protein-repellent dental composite with autonomous crack-healing ability would have a significant impact on the longevity of the composite, which would be reflected in their service lives. However, while these two approaches are successful individually, they have never been evaluated together. In the present study, we report for the first time the design formulation of a dual-loaded dental composite that combines and provides both high protein repellency and self-healing simultaneously in their core and surface.

## 2. Materials and Methods

### 2.1. Chemicals and Reagents

Bisphenol A glycidyl dimethacrylate (Bis-GMA) and triethylene glycol dimethacrylate (TEGDMA) were obtained from Esstech (Essington, PA, USA) and used as received. *N*,*N*-dihydroxy ethyl-*p*-toluidine (DHEPT), ethylene-maleic anhydride (EMA), ammonium chloride, resorcinol, formaldehyde, bovine serum albumin (BSA), camphorquinone (CQ), ethyl 4-(diamethylamino) benzoate (4E), 2-Methacryloylothelxyethyl-phosphorylcholine (MPC), 3 methacryloxypropyltrimethoxysilane, and N-propylamine purchased from Sigma-Aldrich (Saint Louis, MO, USA) were used without further purification. Barium boroaluminosilicate glass particles were obtained from Caulk/ Dentsply (Milford, DE, USA).

### 2.2. Synthesis of Self-Healing Microcapsules

**Figure 1:**
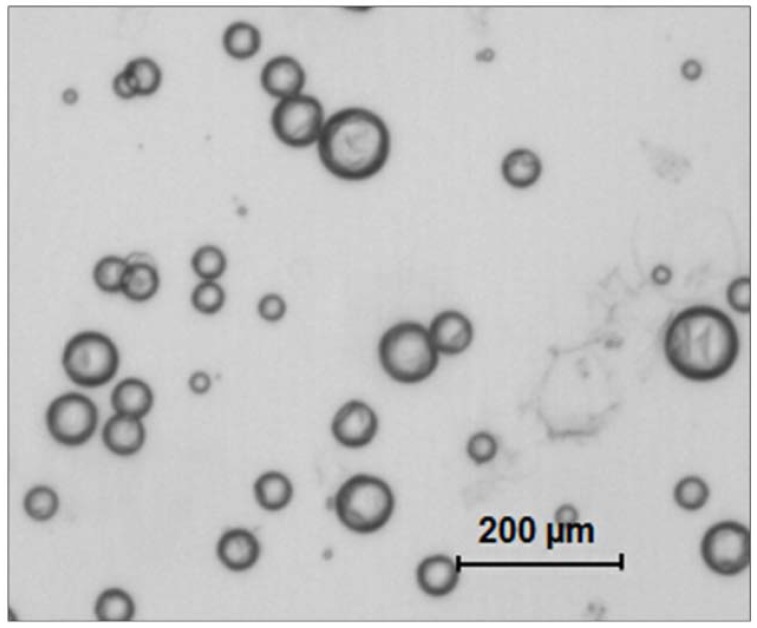
Transmitting optical image of resulting poly (urea-formaldehyde) (PUF) microcapsules loaded with polymerizable TEGDMA and *N*,*N*-dihydroxy ethyl-*p*-toluidine (DHEPT) (an average diameter of 73 ± 31 μm).

Microcapsules (MCS) were prepared using an in-situ polymerization of formaldehyde via the urea method, as described previously [28]. First, DHEPT was added to the TEGDMA monomer at 1 wt %. A mixture of 50 mL of distilled water and 13 mL of a 2.5% aqueous solution of EMA copolymer was prepared in a 250 mL-Erlenmeyer flask. The flask was suspended in a water bath on a hot plate. Second, the shell-forming material urea (1.25 g), ammonium chloride (0.125 g), and resorcinol (0.125 g) were added into the solution under 300 rpm agitation by a magnetic stir bar (Ø = 7.8 mm, length = 50 mm, Fisher Scientific). Resorcinol was added in the reaction of shell formation to enhance the rigidity of the shell [30]. The pH was adjusted to 3.5 by the drop-wise addition of 1 M NaOH. Then, the agitation rate was increased to 400 rpm, and 30 mL of the TEGDMA-DHEPT liquid was added into the flask [31]. After 10 min of agitation, a stabilized emulsion of fine TEGDMA-DHEPT droplets were formed. Then, 3.15 g of a 37% aqueous solution of formaldehyde was added. The stirring was continued with heating to 55 °C for 4 h [28]. In this process, ammonium chloride catalyzed the reaction of urea with formaldehyde to form PUF at the oil–water interface to develop the shell [29]. The resulting microcapsules were rinsed with water and acetone, vacuum filtered, and air dried for 24 h. The microcapsules’ structure was confirmed by optical microscope 4× (TE2000-S, Nikon, Japan) and SEM (Quanta 200, FEI, Hillsboro, OR, USA), as shown in Figure 1.

### 2.3. Concepting MPC and Self-Healing Microcapsules into Composite

A parental composite formulation was made by mixing the following components: Bis-GMA and TEGDMA at a mass ratio of 1:1, 0.2% camphorquinone, 0.8% ethyl 4-*N*,*N*-dimethylamino benzo, and barium boroaluminosilicate glass (mean particle size of 1.4 mm) silanized with 4% 3-methacryloxypropyltrimethoxy silane and 2% n-propylamine. MPC, a methacrylate with a phospholipid polar group in the side chain, was used as the protein-repellent agent. MPC was synthesized according to a reported method [15].

The MPC powder was mixed with photo-activated BisGMA-TEGDMA resin at mass fractions of 7.5%. Then, the composite was then mixed with microcapsules at microcapsule mass fractions of 10%. The chosen mass fractions were selected considering previous investigations of the relationship between the MPC and microcapsules mass fraction and the mechanical properties of the composite [21,28].

### 2.4. Protein Repellence Essay

For the protein adsorption and live/dead essays, each composite paste was placed into disc molds (Ø = 9 mm; 2 mm in thickness). They were light-cured, stored in distilled water at 98.6 °F for 24 h, and sterilized by ethylene oxide, following a previous study [20].

The amount of protein adsorbed on the composite discs was determined by the micro bicinchoninic acid method [32]. Six disks were evaluated for each group. Each disk was immersed in phosphate buffered saline (PBS) for 2 h before immersing in 4.5 g/L bovine serum albumin (BSA) solutions at 37 °C for 2 h. The disks then were rinsed with fresh PBS by stirring method (300 rpm for 5 min). The adsorbed protein was detached in sodium dodecyl sulfate (SDS) 1 wt % in PBS by sonication for 20 min. A protein analysis kit (Micro BCA protein assay kit, Fisher Scientific, Pittsburgh, PA, USA) was used to determine the BSA concentration in the SDS solution. The amount of protein adsorbed on the resin disk surface was calculated from the concentration of protein [22].

### 2.5. Live/Dead Staining of Biofilms

Fluorescence microscopy via live/dead assay was used to directly visualize the early bacterial attachment (4 h after inoculum) over the studied materials. A dental plaque microcosm biofilm model using human saliva was used to promote the biofilm grown over the composites, according to a previous report [33]. The biofilms on the disks were gently washed three times with phosphate-buffered saline (PBS), and then stained using a live/dead bacterial viability kit (Molecular Probes, Eugene, OR, USA). Live bacteria were stained with Syto 9 to produce a green fluorescence, and bacteria with compromised membranes were stained with propidium iodide to produce a red fluorescence [19]. The corresponding images were acquired using appropriate selective filters in the epifluorescence microscope (TE2000-S, Nikon, Melville, NY, USA). The area of green staining (live bacteria) was computed with NIS-Elements imaging software (Nikon, Melville, NY, USA). The area fraction of live bacteria was calculated based on green staining area/total area of the image.

### 2.6. Assessment of Mechanical Properties

#### 2.6.1. Flexural Strength and Elastic Modulus Testing

Following previous studies, composites specimens were placed in metal molds, photo-cured (Triad 2000, Dentsply, York, PA, USA) for 1 min on each side and then smoothed, which produced bar specimens with dimensions of 2 mm× 2 mm× 25 mm (*n* = 6) [29,34]. Twenty-four hours after manufacturing and water storage, specimens were subjected to three-point flexural testing using a computer-controlled Universal Testing Machine—UTM (5500R, MTS, Cary, NC, USA). All tests were performed using a span of 10 mm and a crosshead speed of 1 mm/min. Flexural strength (FS) was measured as FS = 3PmaxL/(2bh^2^), where P max is the load-at-failure, L is a span, b is specimen width, and h is thickness. Elastic modulus (E) was measured as E = (P/d)(L3/[4bh3]), where load P divided by displacement d is the slope in the linear elastic region of the load-displacement curve [34,35].

#### 2.6.2. Fracture Toughness and Self-Healing Assessment

Fracture toughness (K_IC_) was measured using a single edge V-notched beam (SEVNB) method [24]. The SEVNB composite bars specimens for fracture toughness measurement were prepared following a previous protocol [28,29]. After notching the bending bars with a razor blade and 3 µm-diamond suspensions (average notch: depth 700–800 µm; tip radius = 20 µm), the fracture toughness was determined by the same three-point bending test. The photo-cured composites containing the MPC and microcapsules as well the related controls were tested. This yielded the original virgin K_IC_ of the specimens (K_IC-virgin_).

Before testing the self-healing assessment, the two halves of the specimen were attached to the metal mold and placed in a humidor at 37 °C for 24 h. After the fracture, the healing process was trigged [30]. In this period, the disrupted microcapsules released the healing agent TEGDMA-DHEPT, which reacts with the BPO in the resin matrix. The mix of these two components would cause the polymerization of the released liquid to heal and bond the two cracked planes into one cohesive specimen. The sample was fractured again using the same method, and the new fracture toughness (K_IC healed_) was calculated [31]. The self-healing efficiency (ή) was assessed as the percentage of fracture toughness after the healing in comparison with the virgin (ή = K_IC-healed_ K_IC-virgin_ × 100).

### 2.7. Statistical Analysis

Statistical evaluations were performed with SigmaStat 3.5 (Systat, San Jose, CA, USA). The normality distribution of the data and equality of variances were checked using the Kolmogorov–Smirnov test and Levene’s test, respectively. As the data were normally distributed, analysis of variance (ANOVA) and the Tukey test were applied at a significance level of *p <* 0.05.

## 3. Results

**Figure 2:**
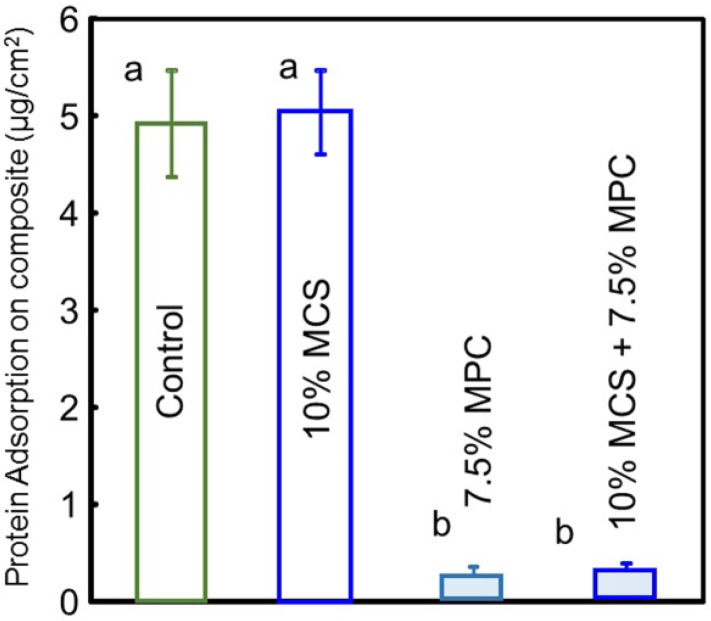
Protein adsorption onto composite surfaces. The composite with 7.5% 2-methacryloyloxyethyl phosphorylcholine (MPC) had the lowest amount of protein adsorption, which was approximately 1/16 those of the composite control and the composite with 10% microcapsules (MCS) (*p* < 0.05). Different letters indicate values that are significantly different from each other (*p* < 0.05).

The amounts of protein adsorption on composite disc surfaces are plotted in Figure 2 (*n* = 6). Adding MPC to composites significantly decreased the protein adsorption (*p* < 0.05). The resin composite with 7.5% MPC had the lowest amount of protein adsorption, which was nearly 5% that of the control and the composite without MPC (*p* < 0.05).

**Figure 3:**
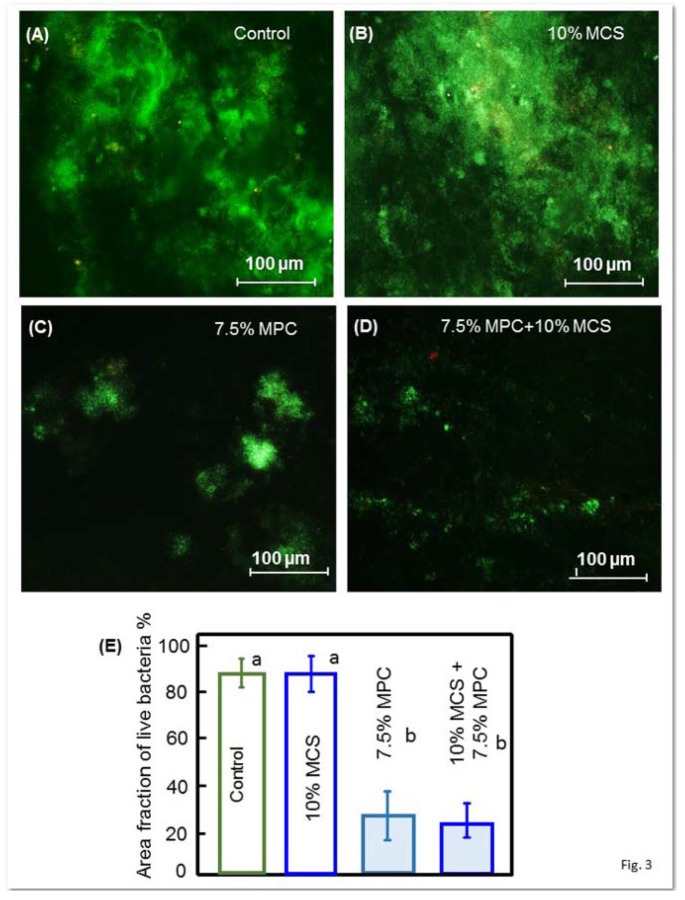
Representative live/dead staining images on the early attachment of oral biofilms of the disks (**A**–**D**). Live bacteria showed green, while dead bacteria showed red, and (**E**) shows the area fraction of the green staining of live bacteria coverage on composite surfaces (mean ± SD; *n* = 6). The composite control had much more bacterial attachment. All groups were covered with live bacteria and few dead bacteria; the images (**C**,**D**) show that the composite with incorporated MPC had noticeably fewer bacteria on the cover zone than the images of composites without MPC (**A**,**B**). Different letters in (**E**) indicate values that are significantly different from each other (*p* < 0.05).

Figure 3 shows the early bacterial attachment onto composite surfaces by representative live/dead staining images after 4 h of the inoculum. Live bacteria were stained green, and dead bacteria were stained red. The composite discs had primarily live bacteria, with few dead bacteria. Both the control composite and the composite with 10% MCS but without MPC had noticeably more bacteria coverage than the composites containing MPC. The quantification of the area fraction of the composite surface covered by live bacteria is plotted in Figure 3E, and corroborates with the outcome found in the qualitative evaluation of images where composites with 7.5% MPC had great bacterial adhesion reduction.

**Figure 4:**
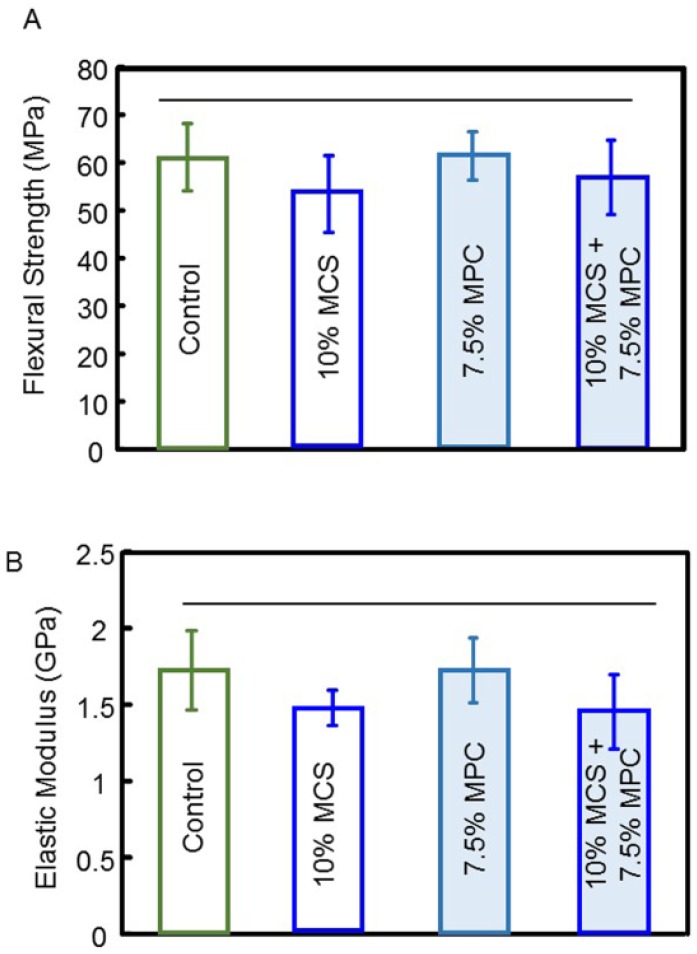
Mechanical properties of resin-containing microcapsules of various concentrations: (**A**) flexural strength, and (**B**) elastic modulus (mean ± SD; *n* = 6). The addition of up to 10% of microcapsules and/or 7.5% MPC resulted in no significant decrease in strength or the elastic modulus of the composite. Horizontal line indicates statistically similar values (*p* > 0.5).

The flexural strength and elastic modulus of composites with combined and isolated tested components were evaluated after one day of water storage, and are plotted in Figure 4 (mean ± SD; *n* = 6). The flexural strength and elastic modulus of a composite containing 10% microcapsules and 7.5% MPC were not significantly different from controls (*p* > 0.5).

**Figure 5:**
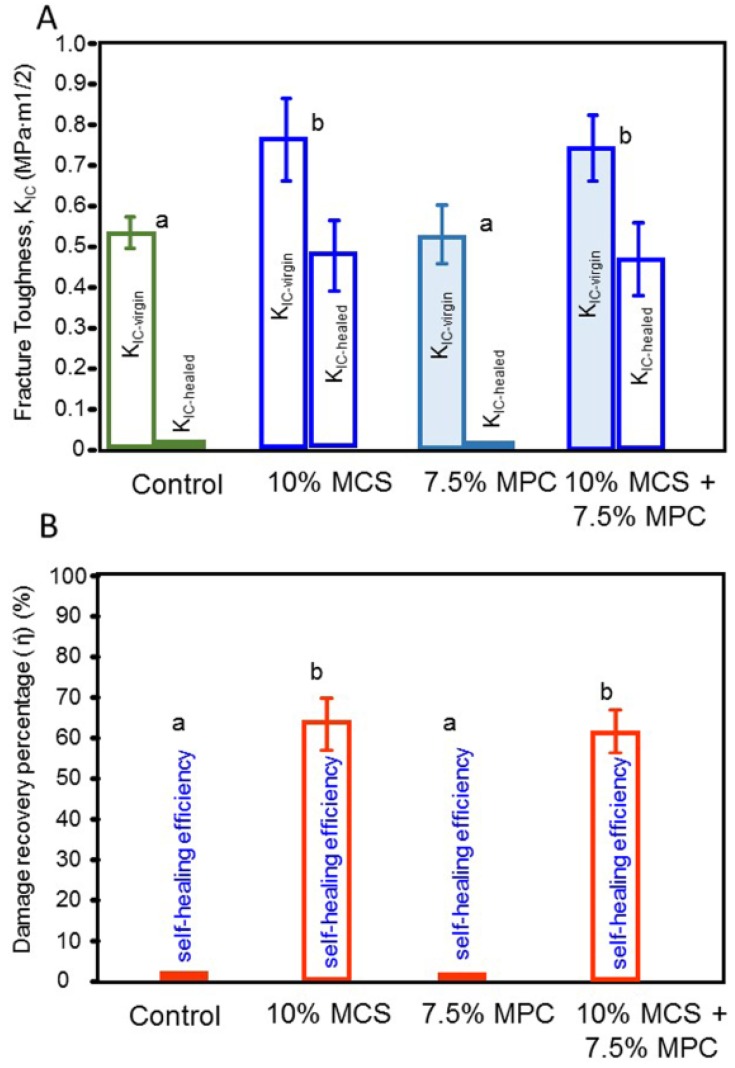
Fracture toughness and self-healing of a composite containing microcapsules and MPC. (**A**) Initial and post-healing fracture toughness (K_IC_), and (**B**) the damage recovery for K_IC_ according to the formulation designed for each tested composite. In each plot, values with dissimilar letters are significantly different from each other (*p* < 0.05).

The critical stress-intensity value, or plane-strain fracture toughness, denoted K_IC_ were expressed in MPa-m^1/2^.The results for all tested composites after one day of water storage are shown in Figure 5. The virgin-healed K_IC_ and the percentage of damage recovery of the composites are expressed for each material. The virgin K_IC_ of the composite to which the microcapsules were added was about 36% higher than that at 0% microcapsules (*p* < 0.05; one-way ANOVA). The healed K_IC_ were similar for the composites containing 10% microcapsules with and without the addition of MPC. The MPC incorporation did not compromise the healed K_IC_ (*p* < 0.05). The self-healing efficiency of 57–71% in damage recovery was obtained in both composites where the microcapsules were present.

## 4. Discussion

Significant advancements in mechanical strength and surface roughness have been achieved in dental composites. Nevertheless, microcracking induced by fatigue and biofilm accumulation are long-standing problems in dental resin composites, and current strategies are non-responsive. In this study, we present an innovative strategy that involves the combination of a protein-repellant approach and the simultaneous targeting healing of the microcracks that eventually lead to the failure of restorations. More specifically, we aimed to design a composite that can fight the recurrence of dental caries around the composite, as well as help elicit mechanisms for the self-correction of many initial micro-cracks without a dentist’s operative intervention. The facets of this approach complement each other for optimal efficacy and increased clinical life service.

The self-healing approach has been investigated in previous reports, which indicate that healing efficiency is relative to the matrix used, and can vary tremendously due to different matrix-healed network interactions [24,30,31]. By concept, the self-healing polymeric materials have the built-in capability to substantially recover their load-transferring ability after damage. Such recovery can occur autonomously or be activated after an application of a particular stimulus (e.g., heat, radiation)[36,37]. Research into producing self-healing dental composites has been based on the release of a healing liquid after cracking produced via fatigue[38]. In the present study, microcapsules with a healing liquid of TEGDMA plus 1% DHEPT surrounded by a PUF shell were used via an in situ polymerization technique in an oil-in-water emulsion [28]. This method allowed the production of microcapsules with an average diameter of 70 ± 24 µm [29], which respond to a massive rupture of the PUF shell when stress is required to propagate a pre-existing flaw. We proposed that this effect was efficient due to sufficient microcapsule stability inside the resin matrix promoted by the roughness of the external surface of microcapsule wall and a suitable thickness of the wall, which also protects the encapsulated healing agent from premature polymerization [29]. Approximately 65% of the original strength was recovered after repair with TEGDMA-DHEPT as a healing liquid. Efficient self-healing has been also demonstrated in previous studies using the applied method of fabrication of microcapsules [29,31]. The microencapsulated healing is quite stable and durable, which broadens the process window for fabricating self-healing composites and prevents the deterioration of the healing capability of the composites during storage [32]. The successful demonstration of this healing system will open pathways for healing in dental composites.

The incorporation of additives with different purposes into a resin matrix would inevitably affect its intrinsic properties. The fraction of each element is critical for the current system to achieve the highest healing efficiency. One major goal of this study was to combine the best performance of those two elements (healing system and protein repellent) without detrimental effects on their highlighted properties. High healing efficiency can be acquired at 10% capsules content so that the fundamental mechanical properties of the matrix are insignificantly affected. Previous studies showed a positive correlation between microcapsule content incorporation and the fracture toughness of the polymer matrix [26,27]. In a previous study, a nearly 40% increase in the original virgin KIC was achieved when the microcapsule mass fraction was increased from 0 to 10%, and slightly reduced when increased to 15% [29]. This finding was also consistent with a previous study showing that the incorporation of up to 6% of microcapsules into a host material did not affect the original flexural strength [30]. Mechanistically, we show that the addition of 10% microcapsules into the composite did not decrease its properties, and can be used as an optimal concentration that reaches up to 70% of healing efficiency. Also, because the healing agent possesses high flow ability and reactivity and belongs to the same family as the matrix polymer, crack healing is automatically conducted at or below room temperature, which offers satisfactory repair effectiveness.

Our results highlight composites for dental restorations that satisfy the characteristics of autonomous recovery after damage and ultra-low protein adsorption, and also, that low bacterial adhesion thereon was successfully achieved. The incorporation of 2-Methacryloylothelxyethyl-phosphorylcholine (MPC) into dental composites displays antibiofilm potential properties regulated by reduced protein adsorption, since the attachment of oral bacteria to a material’s surface is moderated by adsorbed proteins [23]. A recent report has first demonstrated the contribution of MPC as an excellent agent for reducing bacterial adhesion [19]. The present study also confirmed that MPC-containing composite could repel proteins, which indicated that the composite could potentially also reduce biofilm attachment.

The polymer MPC has been shown to be highly hydrophilic and capture the surrounding water that is credited to detach proteins efficiently, thereby repelling protein adsorption [36,37]. Based on this mechanism, it would have the higher mass fraction of MPC in the resin composite to reach the protein-repellent potency. The current study showed for the first time that while the composite containing MPC was indeed strongly protein-repellent, its potency was not compromised via a dual method (MPC & MCS). The addition of MPC at a mass fraction of 7.5% did not adversely affect the mechanical strength of the newly designed material.

The set of tests performed here significantly revealed reduced oral biofilm attachment and a triggered a self-healing response, which prove that our approach was feasible and efficient in vitro, and suggest that this combined strategy may indeed help overcome recurrent caries and material fracture in dental composites.

## 5. Conclusions

This preliminary study demonstrates the feasibility of preparing a self-healing dental composite formulation by embedding the TEGDMA-DHEPT healing agent into microcapsules combined with an antibiofilm surface. High healing efficiency can be acquired at optimal capsules content so that the basic mechanical properties of the matrix are insignificantly affected. In this research, the design and development of a protein-repellent dental composite formulation with autonomous crack-healing ability was successfully performed. The dual strategies for overcoming the most commons problem of composites can provide a new platform for anticaries dental materials.

## References

[1] Ferracane J.L. (2011). Resin composite—State of the art. Dent. Mater..

[2] Ali Z., Eliyas S., Vere J.W. (2015). Choosing the Right Dental Material and Making Sense of the Options: Evidence and Clinical Recommendations. Eur. J. Prosthodont. Restor. Dent..

[3] Krämer N., Reinelt C., Frankenberger R. (2015). Ten-year Clinical Performance of Posterior Resin Composite Restorations. J. Adhes. Dent..

[4] Ástvaldsdóttir Á., Dagerhamn J., van Dijken J.W., Naimi-Akbar A., Sandborgh-Englund G., Tranæus S., Nilsson M. (2015). Longevity of posterior resin composite restorations in adults—A systematic review. J. Dent..

[5] Forss H., Widstrom E. (2004). Reasons for restorative therapy and the longevity of restorations in adults. Acta Odontol. Scand..

[6] Brantley C.F., Bader J.D., Shugars D.A., Nesbit S.P. (1995). Does the cycle of restoration lead to larger restorations?. J. Am. Dent. Assoc..

[7] Beazoglou T., Eklund S., Heffley D., Meiers J., Brown L.J., Bailit H. (2007). Economic impact of regulating the use of amalgam restorations. Public Health Rep..

[8] Demarco F.F., Corrêa M.B., Cenci M.S., Moraes R.R., Opdam N.J. (2012). Longevity of posterior composite restorations: Not only a matter of materials. Dent. Mater..

[9] Chauhan R. (2015). Good short-term survival rates for posterior resin composite restorations. Evid. Based Dent..

[10] Potyrailo R., Rajan K., Stoewe K., Takeuchi I., Chisholm B., Lam H. (2011). Combinatorial and high-throughput screening of materials libraries: A review of state of the art. ACS Comb. Sci..

[11] Cross M.C., Toomey R.G., Gallant N.D. (2016). Protein-surface interactions on stimuli-responsive polymeric biomaterials. Biomed. Mater..

[12] Sibarani J., Takai M., Ishihara K. (2007). Surface modification on microfluidic devices with 2-methacryloyloxyethyl phosphorylcholine polymers for reducing unfavorable protein adsorption. Colloids Surf. B Biointerfaces.

[13] Lewis A.L., Tolhurst L.A., Stratford P.W. (2002). Analysis of a phosphorylcholine-based polymer coating on a coronary stent pre- and post-implantation. Biomaterials.

[14] Huang X.D., Yao K., Zhang H., Huang X.J., Xu Z.K. (2007). Surface modification of silicone intraocular lens by 2-methacryloyloxyethyl phosphoryl-choline binding to reduce Staphylococcus epidermis adherence. Clin. Exp. Ophthalmol..

[15] Ishihara K. (2000). Bioinspired phospholipid polymer biomaterials for making high-performance artificial organs. Sci. Technol. Adv. Mater..

[16] Moro T., Takatori Y., Ishihara K., Konno T., Takigawa Y., Matsushita T., Ung-Il C., Nakamura K., Kawaguchi H. (2004). Surface grafting of artificial joints with a biocompatible polymer for preventing periprosthetic osteolysis. Nat. Mater..

[17] Willis S.L., Court J.L., Redman R.P., Wang J.H., Leppard S.W., Obyrne V.J., Small S.A., Lewis A.L., Jones S.A., Stratford P.W. (2001). A novel phosphorylcholine-coated contact lens for extended wear use. Biomaterials.

[18] Zhang N., Chen C., Weir M.D., Bai Y., Xu H.H. (2015). Antibacterial and protein-repellent orthodontic cement to combat biofilms and white spot lesions. J. Dent..

[19] Zhang N., Ma J., Melo M.A., Weir M.D., Bai Y., Xu H.H. (2015). Protein-repellent and antibacterial dental composite to inhibit biofilms and caries. J. Dent..

[20] Zhang N., Melo M.A., Bai Y., Xu H.H. (2014). A novel protein-repellent dental adhesive containing 2-methacryloyloxyethyl phosphorylcholine. J. Dent..

[21] Zhang N., Zhang K., Melo M.A., Chen C., Fouad A.F., Bai Y., Xu H.H. (2016). Novel protein-repellent and biofilm-repellent orthodontic cement containing 2-methacryloyloxyethyl phosphorylcholine. J. Biomed. Mater. Res. B Appl. Biomater..

[22] Zhang N., Weir M.D., Romberg E., Bai Y., Xu H.H. (2015). Development of novel dental adhesive with double benefits of protein-repellent and antibacterial capabilities. Dent. Mater..

[23] Zhang N., Chen C., Melo M.A., Bai Y.X., Cheng L., Xu H.H. (2015). A novel protein-repellent dental composite containing 2-methacryloyloxyethyl phosphorylcholine. Int. J. Oral Sci..

[24] Huyang G., Debertin A.E., Sun J. (2016). Design and development of self-healing dental composites. Mater. Des..

[25] Jones A.S., Rule J.D., Moore J.S., Sottos N.R., White S.R. (2007). Life extension of self-healing polymers with rapidly growing fatigue cracks. J. R. Soc. Interface.

[26] Jones A.S., Rule J.D., Moore J.S., White S.R., Sottos N.R. (2006). Catalyst morphology and dissolution kinetics of self-healing polymers. Chem. Mater..

[27] Dry C. (1996). Procedures developed for self-repair of polymer matrix composite materials. Compos. Struct..

[28] Wu J., Weir M.D., Melo M.S., Zhang Q., Zhou C., Xu H.H. (2016). Novel self-healing dental resin with microcapsules of polymerizable trimethylene glycol dimethacrylate and *N*,*N*-dihydroxy ethyl-*p*-toluidine. Dent. Mater..

[29] Wu J., Weir M.D., Melo M.S., Xu H.H. (2015). Development of novel self-healing and antibacterial dental composite containing calcium phosphate nanoparticles. J. Dent..

[30] Brown E.N., Kessler M.R., Sottos N.R., White S.R. (2003). In situ poly (urea-formaldehyde) microencapsulation of dicyclopentadiene. J. Microencapsul..

[31] Hillewaerea X.K.D., Preza F.E.D. (2015). Fifteen chemistries for autonomous external self-healing polymers and composites. Prog. Polym. Sci..

[32] Wu J., Weir M.D., Melo M.A., Strassler H.E., Xu H.H. (2016). Effects of water-aging on self-healing dental composite containing microcapsules. J. Dent..

[33] Melo M.A., Cheng L., Weir M.D., Hsia R.C., Rodrigues L.K., Xu H.H. (2013). A novel dental adhesive containing antibacterial agents and calcium phosphate nanoparticles. J. Biomed. Mater. Res. B Appl. Biomater..

[34] Xu H.H., Moreau J.L., Sun L., Chow L.C. (2010). Novel CaF(2) nanocomposite with high strength and fluoride ion release. J. Dent. Res..

[35] O’Donnell J.N., Schumacher G.E., Antonucci J.M., Skrtic D. (2009). Adhesion of amorphous calcium phosphate composites bonded to dentin: A study in failure modality. J. Biomed. Mater. Res. B Appl. Biomater..

[36] Tateishi T., Kyomoto M., Kakinoki S., Yamaoka T., Ishihara K. (2014). Reduced platelets and bacteria adhesion on poly (ether ether ketone) by photoinduced and self-initiated graft polymerization of 2-methacryloyloxyethyl phosphorylcholine. J. Biomed. Mater. Res. A.

[37] Goda T., Konno T., Takai M., Ishihara K. (2007). Photoinduced phospholipid polymer grafting on Parylene film: Advanced lubrication and anti-biofouling properties. Colloids Surf. B Biointerfaces.

[38] Wu D.Y., Meurer S., Solomon D. (2008). Self-healing polymeric materials: A review of recent developments. Prog. Polym. Sci..

